# The impact of climatic factors on tick-related hospital visits and borreliosis incidence rates in European Russia

**DOI:** 10.1371/journal.pone.0269846

**Published:** 2022-07-20

**Authors:** Pantelis Georgiades, Ekaterina Ezhova, Meri Räty, Dmitry Orlov, Markku Kulmala, Jos Lelieveld, Svetlana Malkhazova, Kamil Erguler, Tuukka Petäjä

**Affiliations:** 1 Environmental Predictions Department, Climate and Atmosphere Research Centre, The Cyprus Institute, Nicosia, Cyprus; 2 Institute for Atmospheric and Earth System Research (INAR), Faculty of Science, University of Helsinki, Helsinki, Finland; 3 Department of Biogeography, Faculty of Geography, Lomonosov Moscow State University, Moscow, Russia; 4 Max Planck Institute for Chemistry, Mainz, Germany; Tufts University Cummings School of Veterinary Medicine, UNITED STATES

## Abstract

Tick-borne diseases are among the challenges associated with warming climate. Many studies predict, and already note, expansion of ticks’ habitats to the north, bringing previously non-endemic diseases, such as borreliosis and encephalitis, to the new areas. In addition, higher temperatures accelerate phases of ticks’ development in areas where ticks have established populations. Earlier works have shown that meteorological parameters, such as temperature and humidity influence ticks’ survival and define their areas of habitat. Here, we study the link between climatic parameters and tick-related hospital visits as well as borreliosis incidence rates focusing on European Russia. We have used yearly incidence rates of borreliosis spanning a period of 20 years (1997-2016) and weekly tick-related hospital visits spanning two years (2018-2019). We identify regions in Russia characterized by similar dynamics of incidence rates and dominating tick species. For each cluster, we find a set of climatic parameters that are significantly correlated with the incidence rates, though a linear regression approach using exclusively climatic parameters to incidence prediction was less than 50% effective. On a weekly timescale, we find correlations of different climatic parameters with hospital visits. Finally, we trained two long short-term memory neural network models to project the tick-related hospital visits until the end of the century, under the RCP8.5 climate scenario, and present our findings in the evolution of the tick season length for different regions in Russia. Our results show that the regions with an expected increase in both tick season length and borreliosis incidence rates are located in the southern forested areas of European Russia. Oppositely, our projections suggest no prolongation of the tick season length in the northern areas with already established tick population.

## 1 Introduction

Ticks species of *Ixodes* genus are the most important vectors of dangerous tick-borne diseases, such as encephalitis and borreliosis, in the Northern hemisphere [1]. In Europe and Russia, the most widespread species are *Ixodes ricinus* and *Ixodes persulcatus*, commonly known as the castor bean tick and taiga tick, respectively. Both species have a similar four-stage life cycle, which consists of the egg, larva, nymph and adult stages, and a life span between three and six years [2]. The ticks are active during the warm season, and they enter a diapause state in the winter. This behavioral diapause is possible due to the ability of the ticks to perceive changes in day length and environmental factors (e.g., humidity and temperature), and allows for safe overwintering at any stage except the egg stage for *I. persulcatus* [3, 4]. Numerous studies focus on the connections between environmental factors and ticks’ abundance and questing activity [1, 5–9]. Based on studies carried out using data originating from Russia, when periods with mean daily temperatures above 10°C are considered, the habitat of *I.*p*ersulcatus* was found to be limited by the minimum accumulation of 1410°C days, whereas the development of *I. ricinus* required a minimum of 1460–1550°C days [1, 10]. Another climatic parameter, influencing ticks’ abundance is humidity, which is represented either by accumulated precipitation or by the hydrothermal index, i.e., the ratio between the accumulated precipitation and temperature sums. Hydrothermal indices and accumulated precipitation optimal for ticks of different species are dependent on the temperature sums and they are reported by e.g., Balashov (1998), Sirotkin and Korenberg (2018) [1, 2]. In Europe (Sweden), the population of ticks *I. ricinus* was not established in the areas where the snow cover period was longer than 150 days and the length of vegetation period was below 170 days [11].

As the probability of ticks’ survival is strongly dependent on environmental factors, climate change can drive tick-borne diseases to the north [3, 12]. Recent studies report increases in *Ixodes* tick activity and tick-borne diseases in the northern areas previously not inhabited by ticks. These trends have been reported for Sweden [13], Norway [14, 15], Iceland [16], USA [17], Canada [18] and Russia [19–21]. In Sweden, climate change and the associated rise in temperature and the increased population of deer are most likely driving the recorded range expansion of tick-borne diseases [13]. Similarly, rising temperatures and the dispersion of ticks of animal hosts were suggested as the main driving factors in other countries.

Many studies have attempted to link the northwards propagation of ticks to warming climate, and have identified a correclation between the increasing temperature trends and the incidence rates of tick bites. Tokarevich et al. (2011) reported a significant strong correlation between the mean annual air temperature and encephalitis incidence rates in Arkhangelsk region for the time period between 1980 and 2007 [19]. A similar growing trend was also observed in the Komi Republic, for both the mean annual air temperature and encephalitis incidence rates for a period of forty two years [20]. A recent study focusing on the Sakha Republic in Eastern Siberia reported a significant correlation between the tick bite rates with the mean annual air temperature, average temperature in the coldest month of the year and hydrothermal coefficient [21]. However, other studies criticize the approach solely based on the climatic variables and emphasize the importance of other factors driving ecosystem changes such as urbanization, change in land use and forest fires [1].

Although modelling approaches, which account for the ticks’ life cycle, host dynamics and land use change, and take into consideration the complexity of vector dynamics and vector-host relationships, have been previously used in a number of studies [22, 23], it remains a challenging task due to the poor availability of reliable data and are prone to over-parameterization due to their complexity [12]. Other studies make predictions based on a reduced set of parameters, mainly including climatic variables [12]. Even though these studies have employed an incomplete set of parameters [24, 25], their results are, nevertheless, useful and can be compared to the output of more advanced models when the latter becomes accepted by the scientific community.

This study aims to assess the link between climatic parameters and tick bites as well as borreliosis incidence rate for European Russia. In contrast to previous studies, we do not consider spatial propagation of ticks to new sites. Instead, we aim to predict the temporal evolution of tick-related visits and incidence rates in the territories already inhabited by ticks. We focus mainly on forested areas within the latitudes from 52°N to 65°N. Instead of considering separate regions, we show that the regions can be clustered based on the temporal dependencies of incidence rates pertaining to areas with a certain tick species’ abundance. Furthermore, we use multiple linear regression method to identify the most important climatic factors influencing borreliosis incidence rates for different clusters of regions. Finally, we use deep learning methods to simulate the length of the tick season and make prognosis for the RCP8.5 climatic scenario (‘business as usual’). We postulate that the tick season length is indicative of the risks associated with tick borne diseases in the changing climate until the end of the century.

## 2 Description of data sets and data analysis methods

We chose European Russia because the regions are relatively small compared to those in the Asian part, which allows for higher spatial resolution. In addition, European Russia is a sympatric zone for both types of ticks, *I. ricinus* and *I. persulcatus*. In Western Europe, *I. ricinus* is the dominating species although the first evidence of establishing populations of *I. persulcatus* in Finland and Sweden has been recently reported [26, 27]. In Siberia, oppositely, the only species found is *I. persulcatus*.

### 2.1 Medical data set

The medical data used in this study originate from the Rospotrebnadzor’s (Russian Federal Service for Surveillance on Consumer Rights Protection and Human Wellbeing) official statistical database on Ixodid tick-borne borreliosis (Lyme borreliosis) incidence rates per 100 000 population in European part of Russia for the 20-year period of 1997 to 2016. The incidence rates are presented for federal subjects of European Russia. Rospotrebnadzor is responsible for the investigation of outbreaks and cases of infectious diseases at all levels of medical care, the inspection of laboratory diagnostics and the routine monitoring of the epidemiological situation (as well as of natural foci of zoonotic infections), and the monitoring and assessment of food, air and water quality. The procedure in which such cases are reported is that an initial emergency notification is sent to the epidemiological department of the regional Rospotrebnadzor by the medical practitioner when a patient is suspected to have an infectious disease at any level of medical care. This is then confirmed or excluded from the database based on the results of the specific laboratory tests and the diagnosis is logged by Rospotrebnadzor accordingly. Rospotrebnadzor provides such reports on infectious diseases on a regular basis, which are published and freely accessed through its regional and federal websites [28].

For the purposes of this study, the medical data was retrieved from the Rospotrebnadzor database and provided to us by the Moscow State University, Department of Medical Geography. It includes annual borreliosis incidence rates per 100 000 population for the federal regions of European Russia for a 20-year period (1997–2016). In addition, we used the data set on the number of weekly visits to hospitals for each region or subject: weekly number of patients with tick bites during the tick season. The latter data set is available for 2 years, 2018 and 2019.

### 2.2 Meteorological data sets

The meteorological station data include daily measurements of temperature at 2m, precipitation and snow depth for 1–9 stations per federal region for the period between 1997 and 2016. The list of stations is provided in S1 Table. The data set is openly available from Roshydromet data portal meteo.ru (last access 22.02.2020) and a description of the data sets is provided by [29].

To capture the spatio-temporal variability of the weather in the European Russia, we obtained meteorological data through the ERA5 atmospheric reanalysis for the global climate dataset from the Copernicus Data Store (CDS) [30], using the CDS Toolbox API. The variables used in this study are shown in S2 Table. The dataset was retrieved in netcdf format and had an hourly temporal resolution. Spatial and temporal interpolations of the dataset were performed using the xarray module in Python 3.8.

### 2.3 Meteorological dataset for the future projections (NASA-NEX-GDDP, CMIP5)

For training the neural networks and performing the future projections of tick-related hospital visits, an ensemble dataset comprised of six bias-adjusted and statistically downscaled NASA-NEX-GDDP climate datasets CMIP5 simulation models (CCSM4, CNRM-CM5, GFDL-ESM2G, MPI-ESM-LR, MRI-CGCM3, CSIRO-Mk3–6-0) (RCP8.5 scenario) was used [31, 32]. The datasets are available with a spatial resolution of 0.25° (720x1440 lat-lon regular grid) and daily temporal resolution. The datasets were averaged to match the weekly temporal resolution of the borreliosis incidence data using Python 3.8 and the xarray module. The variables used from the CMIP5 models are shown in S3 Table.

### 2.4 Ancillary spatially resolved dataset (photoperiod)

The day length (time between sunrise and sunset) was calculated using the Brock model [33]. Brock defines daylength at the point where the center of the sun is even with the horizon. The declination of the Earth is calculated by [34]:

$$\begin{array}{c} \phi =23.45*sin(\frac{283+J}{265}) \end{array}$$ (1)

where J is the day of the year. The sunrise/sunset hour-angle is calculated as:

$$\begin{array}{c} hourAngle=cos^{-1}\left(-tan\left(L\right)tan\left(\phi \right)\right) \end{array}$$ (2)

where L is the latitude. Finally, day length (D) is calculated by:

$$\begin{array}{c} D=2*\frac{hourAngle}{15} \end{array}$$ (3)

The photoperiod was calculated using the method described above, for a 0.25° regular grid of the areas enclosed by the clusters used in this study. The centre of each grid box was used in the calculations and the average day length for each federal region was calculated by averaging the day length of all the grid boxes enclosed by it for each day. Finally, the weekly average photoperiod was calculated, which was used in the training and projection procedures by the deep learning models.

### 2.5 Linking annual incidence rates and meteorological parameters

As a first step in this study, we aimed to identify potential links between the borreliosis incidence rates and the meteorological parameters, which are presented in Table 1. For this purpose, the mean for each region was calculated using the available data from the meteorological stations in each of them. Flagged data points were ignored in these calculations.

**Table 1:** Parameters used in linear regression analysis. The description was omitted for self-descriptive parameters. Winter parameters are calculated for the whole winter preceding the tick season with the observed borreliosis incidence rates.

Parameter	Description
Length of the active tick season	From beginning of first 7 consecutive days in spring with daily mean temperatures of at least 5°C to last day before first 7 consecutive days in autumn with daily mean temperatures below 5°C
Median temperature of the active tick season	
Sum of temperatures during the active tick season	Sum of daily mean temperatures over all days in active tick season
Freezing degree-days	Sum of daily mean temperatures over all days in November-March with daily mean temperature below 0°C
Mean temperature of the coldest 10% of winter days (in November-March)	
Precipitation during the active tick season	Accumulated precipitation over all days in active tick season
Hydrothermal index	Sum of accumulated precipitation divided by sum of temperatures on days with temperatures exceeding 10°C
Number of days with detected snow cover	
Number of days with snow cover depth exceeding 10 cm	
Number of days with snow cover depth exceeding 20 cm	
Median snow cover depth	
Date of snowmelt	Julian day following last day with snow cover less than 4 days apart from previous day with snow
Freezing degree-days without snow	Sum of daily mean temperatures over all days with daily mean temperature below 0°C and without detected snow cover

First, we made an effort to identify any linear correlations between incidence rates and meteorological parameters in different regions separately and for clusters of regions. Although this procedure revealed potentially important parameters for each cluster (i.e., increase or decrease of incidence rates with some parameters), none of these dependencies was statistically significant.

We adapted our approach, as both incidence rates and meteorological parameters within the clusters were reasonably close to each other. To reduce the fluctuations in the data sets, we calculated the median values of incidence rates and meteorological parameters for each year and each cluster of data. Then we applied a multiple linear regression for each cluster using the standard Matlab package. For two clusters out of three, the results for a number of parameters were marginally significant, with p-value close to 0.1. In these data sets, we identified outliers based on the residuals and removed one outlier from each data set. In both cases the latter procedure improved the model and made the corresponding dependencies statistically significant.

### 2.6 Time lagged cross-correlation (TLCC)

To establish the similarity between the borreliosis related weekly hospital visits and the environmental and the climatic features described above, we used a time lagged cross-correlation approach. The time series of the weekly hospital visits and each of the ERA5 and CMIP5 variables were considered as two signals, which were shifted in time relative to each other. The cross-correlation coefficient was calculated at each time shift (lag time), to compare the two signals and investigate any periodicities in the data. In the case that the data is periodic, the correlation coefficient with lag curve is expected to have a positive peak when the two signals are in phase and a negative peak when the two signals are out of phase. This type of analysis can be used to establish a leader-follower relationship between the two time-series’ and, in this case, to gauge whether the climatic/environmental variables used contained useful information to be included in a predictive model.

### 2.7 Long short-term memory (LSTM) neural networks

A long short-term memory (LSTM), a special kind of recurrent neural network (RNN) was chosen to be trained to project the weekly tick related hospital visits. An LSTM network consists of a pre-defined number of memory cells, which are each comprised of an input gate, an output gate and a forget gate. This kind of configuration allows the neural network to store information over extended time intervals and, thus, regulate the flow of information in and out of the cell [35, 36]. Furthermore, LSTM networks have been demonstrated to outperform traditional learning algorithms and feed-forward neural networks in tasks like time-series classification, image and audio processing and anomaly detection [37]. Due to the demonstrated performance advantage of LSTM networks in time-series classification problems, this kind of models were chosen instead of traditional machine learning classifiers or statistical time-series models, like ARIMA. Furthermore, the ability of LTSM networks to remember data, allows it to consider long-term environmental dependencies, something that other machine learning methods lack. All the data management, data manipulation, training and projection procedures were performed in Python 3.8 with extensive use of the tensorflow framework and the pandas, NumPy and scikit-learn modules.

#### 2.7.1 LSTM model architecture and training procedures

Two independent neural networks were trained, one for each of the clusters in which a distinct species of tick is found (the Northern and Southern clusters). Both neural networks shared the same architecture, which consisted of 32 LSTM units and an output layer with shape (4, 1). To minimise overfitting, a number of measures were taken, such as implementing a dropout and a recurrent dropout with a 0.25 rate, and applying L2 kernel, recurrent and bias regularisation and a batch normalisation layer. In addition, during training, an early stopping callback function was implemented, in which the training loss was monitored, and training was interrupted when the metric did not improve after 150 epochs.

For training the neural network, the input features were scaled using the StandardScaler method, included in the scikit-learn module. The features used for training and projecting tick related hospital visits include the climate variables from the CMIP5 models (average, minimum, maximum and diurnal temperature, and precipitation) and photoperiod. Furthermore, the weekly hospital visits for each region were re-scaled into a 0–100 range and binned into 4 classes, as shown in Table 2. In addition, the four classes were transformed into categorical classes. As the quantity of data was limited, a train-test split was omitted and instead it was decided to train the two independent models for the Northern and Southern regions independently and use the mixed regions as an evaluation dataset.

**Table 2:** Classification of normalised hospital visits used for training the LSTM neural network.

Normalised hospital visits	Class no.
0	0
0–33	1
33–66	2
66–100	3

To account for the delayed effect of climate variables, such as temperature and precipitation, to the life cycle of the ticks, the feature set of the previous two weeks were included for each week’s prediction (time step = 2). Furthermore, the time series for each federal region was treated independently in training. For each cluster of federal units (northern and southern cluster), the neural network’s weights were randomly initialised and trained on the time-series data of the federal regions belonging to the respective cluster sequentially. After model initialization, the training of the neural network commenced on the first federal region of the cluster, using the Adam optimizer, while the accuracy of the model was monitored in terms of the categorical cross-entropy accuracy. A schematic representation of the training and projection procedures for each cluster of federal regions is shown in S1 Fig. This carried on until the callback function was triggered, which paused the training procedure, until the time-series of the next federal region was loaded, and training continued. This procedure carried on, until all the federal regions for each cluster were used. After the procedure looped through all the federal regions in each cluster, the LSTM models’ weights were frozen and the model was saved, to be used at a later stage for projecting hospital visits.

## 3 Results

### 3.1 Splitting regions in accordance with the distribution and life cycles of the tick species

*Ixodes ricinus* habits light mixed and deciduous forests and associated areas. However, the longitudinal span of the habitat of this species is rather wide: from Karelia in the north to Ukraine in the south [38]. The eastern boundary is in the European part of Russia, and it has quite a complex shape due to historical reasons [38]. In the west, the habitat of *I. ricinus* includes Europe.

The areas of habitat of *Ixodes persulcatus* include the middle and southern taiga and span from the east to the west over all the Russia [38]. In European Russia, this area is bounded by the 62–63°N parallel in the north and the 53–54°N parallel in the south.

**Fig 1:**
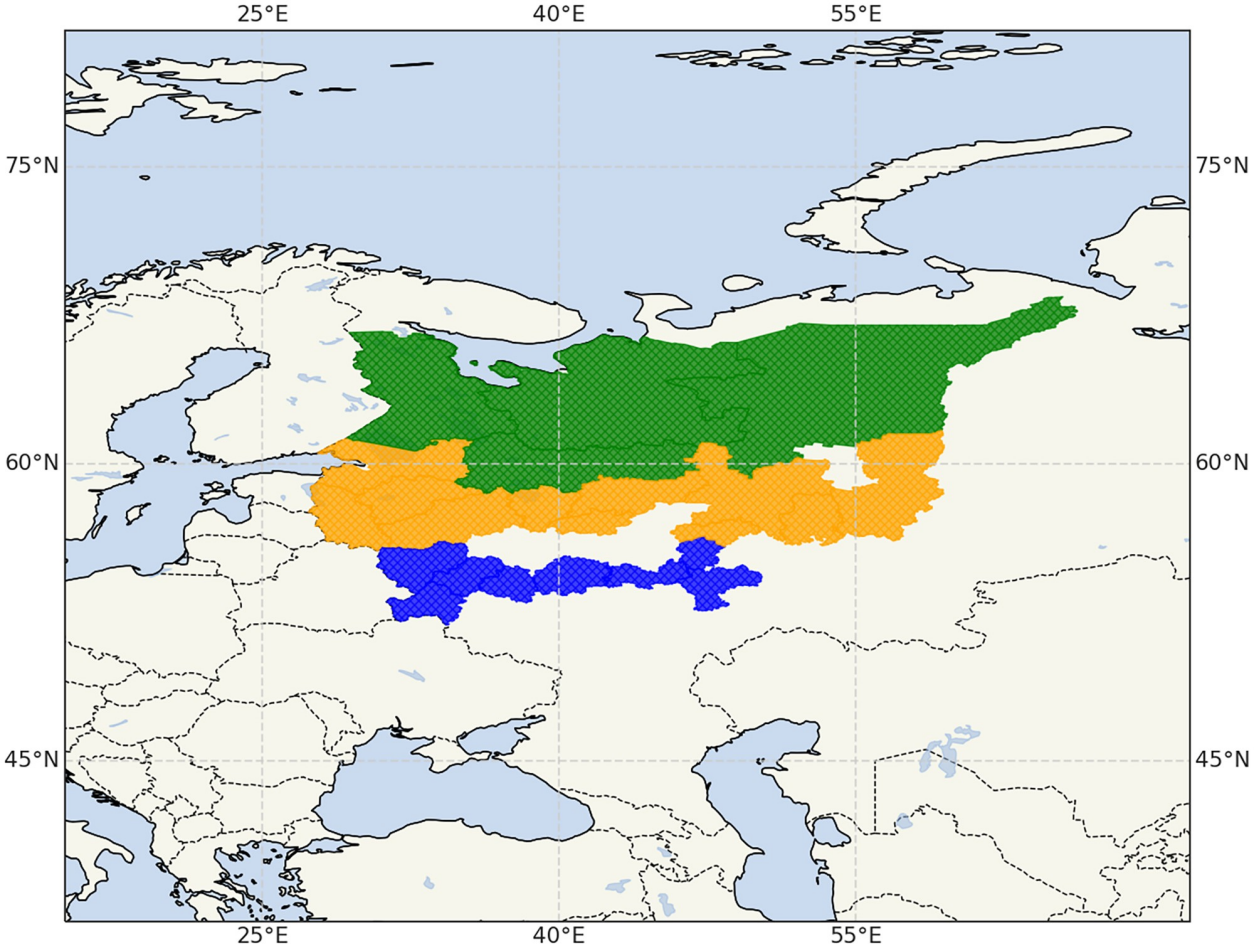
The geographical distribution of the clusters created based on the tick species found in them. In green, the Northern cluster of federal regions, where *I. persulcatus* dominates the tick population and in blue, the Southern federal regions where the *I. ricinus* dominates the tick population. Lastly, the federal regions where both species are found are shown in orange. Voids between the clusters mark the regions where data sets on hospital visits are not available. The map was created using Cartopy in Python and the Natural Earth raster and vector map data, which are freely available through the public domain [39].

The areas of habitat determine different tick distribution over the regions located in different vegetation zones. For example, *I. persulcatus* is a dominating species in the northern federal regions and *I. ricinus* is a dominating species of the two in the most southern federal regions within the habitat of these two species, as shown in Fig 1. Therefore, we used the data sets on weekly hospital visits and annual incidence rates to cluster the distinct types of regions. We normalized the annual data sets with respect to the regional mean values.

**Fig 2:**
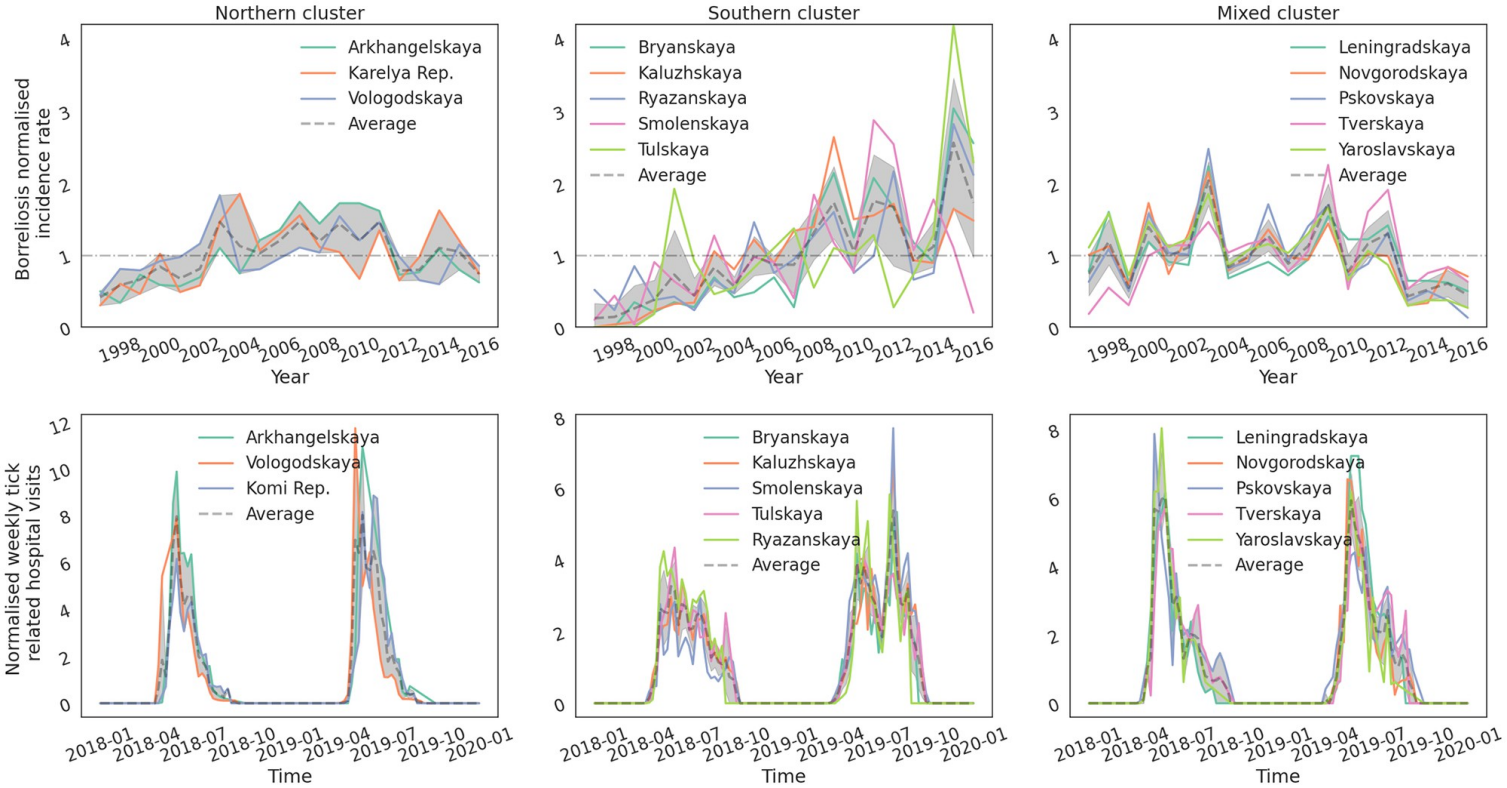
The yearly normalised (with respect to the mean) borreliosis incidence rates for the federal regions investigated (top panels) and normalised (with respect to the mean) weekly tick related hospital visits (bottom row of panels). The Northern cluster of federal regions is shown on the left column of panels, the Southern cluster in the middle column and the mixed cluster on the right column. The dashed line shows the average incidence rate for the federal regions in each region, whereas the dash-dot line indicates the mean for each cluster.

In Fig 2, we present the normalised (with respect to the mean) annual incidence rate of borreliosis and the weekly tick-related hospital visits for the three clusters of federal regions investigated in this study. The normalised annual incidence rates in the Northern cluster of regions, with the tick population dominated by *I. persulcatus* (Arkhangelsk, Karelia, Vologda), demonstrate the following pattern: doubling incidence rates from the half mean value to the mean value during 1997–2003. The weekly hospital visits have one strong peak in May-June, which corresponds to one stage in the tick’s life cycle. Ticks can attack human in two stages of their development cycle, nymph and imago. In the northern latitudes, the ticks can pass only one stage of their development before they diapause or die.

In the Southern cluster of federal regions, where the tick population is dominated by *I. ricinus* (Bryansk, Kaluga, Ryazan, Smolensk and Tula), the normalised annual incidence rates have been increasing from almost 0 to 1.5 mean value during the period 1997–2016. In the weekly tick-related hospital visits time-series, there are two well-pronounced peaks observed, one in spring (May-June) and one in autumn (August-September). In warmer climates, ticks can go through two stages of development during one year, which can explain the two peaks observed during one season.

Finally, in the mixed cluster of federal regions, where both ticks’ species are abundantly present (Leningradskaya, Novgorod, Pskov, Tver, Yaroslavl), the annual normalised incidence rates are fluctuating around the mean value, with some tendency to decrease in the latter part of the time series (2013–2016). The weekly hospital visits time-series have a strong peak in spring (May-June) with a smaller peak in autumn (August-September).

### 3.2 Annual incidence rates vs local meteorological parameters

The results of the multiple linear regression analysis performed on the three identified clusters’ incidence rates and climatic/environmental parameters is summarised in Table 3. As shown, the parameters which were identified as having statistically significant correlation with the incidence rates were different for each of the clusters. In the Northern cluster, the only parameter which was found to be statistically significant was the date of snowmelt, whereas in the mixed cluster the identified parameters were the median temperature of the active tick season and the hydrothermal index. Lastly, in the Southern cluster, only the accumulated heat during the growing season was found to be significantly correlated with the incidence rates.

**Table 3:** The results of multiple linear regression analysis for different clusters of regions. The regions with one removed outlier are written in italic.

Regions	Parameters significantly correlated with incidence rates	Regression	R	p-value
*North*	Snow melt date	IR = 3.632—0.020 meltDay	0.49	0.04
Mixed	Median temperature of the tick season, hydrothermal index	IR = 2.272—0.159 medTemp + 6.117 hy_idx	0.63	0.01
*South*	Temperature sum during the active tick season	IR = 0.778 +0.0005 TSum	0.52	0.02

The association between the snowmelt date and borreliosis incidence rates in the North is possibly related to human behaviour. In the weekly tick-related hospital visits time series dataset for the years 2018 and 2019, shown in Fig 2, there is a peak observed in the first week of May, which is also a holiday week in Russia when many people choose to move to the countryside, and, thus, increase their exposure to potential tick bites. In addition, our analysis shows that the snow-melt date has shifted by approximately two weeks earlier in the year, from day ∼140 to day ∼125. This could potentially facilitate better weather conditions during the holiday weeks and, hence, increase the number of people visiting parks and forests.

In the Mixed cluster, the association with temperature is weaker than that with the hydrothermal index. In addition, there were no discernible trends observed in the incidence rates, as it fluctuates around the mean value. Our results predict some decrease of incidence rates with growing temperature. This may be considered as counter intuitive, since higher temperatures typically favor the development of ticks at all stages. Presumably during the warmer tick season, the prevailing species is *I. ricinus*, which is less contaminated by borrelia [40]. The link to hydrothermal index points at the importance of moisture availability and the balance between temperature and humidity. The median index in the Mixed cluster changed between 0.12 and 0.20.

In the Southern cluster, the strongest link found was with the temperature sum parameter, which should not be surprising, given the observed strong secondary peak in autumn in hospital visits. A prolonged warm season facilitates tick activity, which has been also demonstrated for *I. ricinus* in other studies. Interestingly, adding winter parameters, such as snow cover and freezing degree-days, which also showed some potential importance in the preliminary analysis, did not improve the correlation. Freezing degree-days is likely not a fully independent parameter from the temperature sums during the active season.

Even though a number of climatic parameters were identified to have significant moderate correlations with annual borreliosis incidence rates in the three clusters, using exclusively climatic parameters in linear regression models resulted in predictive performance less than 50%. This is in line with previous studies of the relation of tick-borne encephalitis in the Baltics to climate change, which proposed that climate alone cannot explain the observed upsurge in the late 20^th^ century [41]. The drivers for this change is probably a synergy of factors, including climate and other human-driven contributors, like land-use and human behaviour, e.g. increase of temperature and lengthening of the summer season can potentially lead to more people doing outdoor activities and, thus, contribute to a higher rate of incidence of tick-borne diseases.

### 3.3 Results from the Time Lagged Cross Correlation (TLCC) analysis

To investigate any delayed effects of climate and environmental variables to the incidence of tick related hospital visits, we used a time lagged cross-correlation analysis approach. Here, the time-series of the weekly tick-related hospital visits and climate/environmental variables were considered as two signals, which were shifted in increments of 1 week between −20 and +20 weeks and their cross-correlation coefficient was calculated at each step.

**Fig 3:**
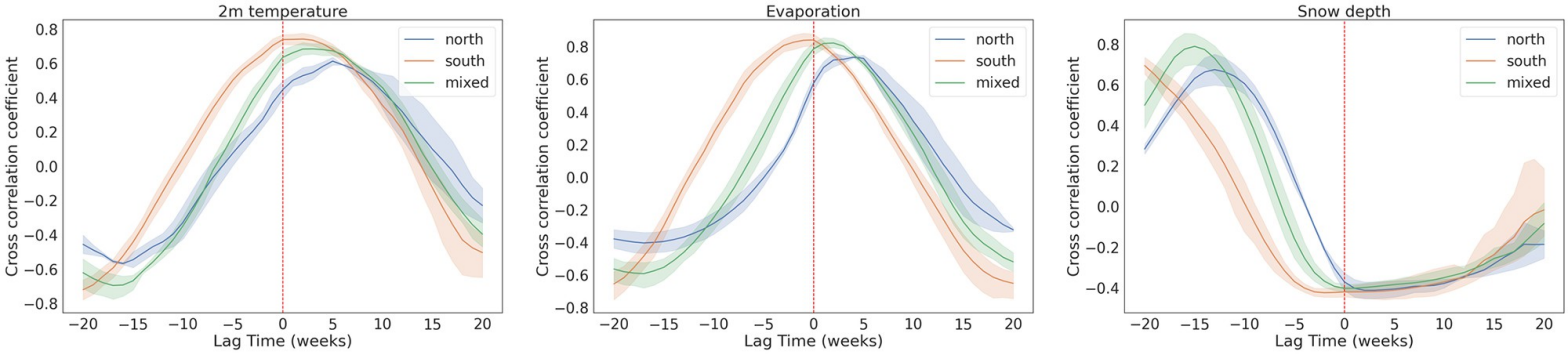
Time lagged cross correlation analysis for the temperature at 2m (left panel), evaporation (middle panel) and snow depth (right panel) variables with respect to the weekly hospital visits due to tick bites. Each panel shows the averaged TLCC curve for the three clusters of federal clusters, according to the tick species inhabiting them, investigated in this study.

In Fig 3, we present the time lagged cross-correlation analysis between the temperature at 2m, evaporation and snow depth variables and the weekly tick-related hospital visits for the three clusters of federal regions investigated. In the Southern cluster, the temperature and evaporation variables are observed to have a cross-correlation coefficient peak around zero lag time, as the peaks of the two series occur around the same time of the year (S2 and S3 Figs). In the Northern and mixed clusters of federal regions, the tick-related hospital visits time series precedes the temperature and evaporation curve by 2–3 weeks (mixed cluster) and 4–6 weeks (Northern cluster). In all three clusters, there is a significant lag around 10–15 weeks between the snow depth variable and the hospital visits time series, as expected due to the biology of both tick species’ (S4 Fig). Ticks are in a diapausing state during harsh winter conditions; thus, no tick bites are expected during these times.

**Fig 4:**
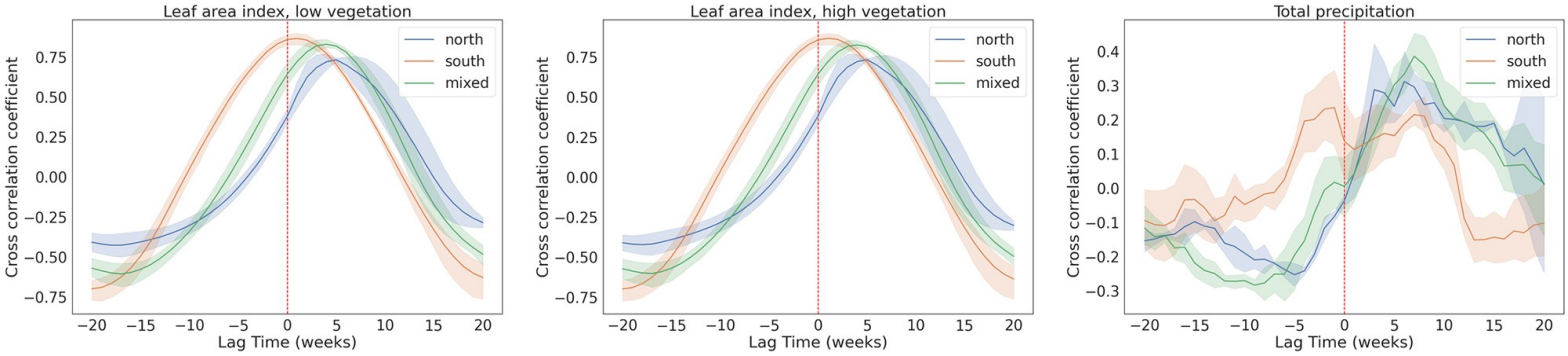
TLCC analysis for the low (left panel) and high (middle panel) vegetation indices and precipitation (right panel) variables with respect to the weekly tick-related hospital visits for the three federal region clusters investigated in this study. Each panel shows the averaged TLCC curve for the three clusters.

The low and high vegetation indices as well as the precipitation TLCC analysis with respect to the weekly tick-related hospital visits are presented in Fig 4. The two vegetation indices had the peak cross-correlation coefficient with the hospital visits time series around zero lag time for the Southern cluster, whereas it lagged by 2–3 weeks in the mixed cluster and 4–6 weeks in the Northern cluster. There was no significant correlation observed at any lag time for the precipitation time-series for any of the three clusters (S5 Fig). Other variables, like skin temperature and snow related variables, had similar TLCC curves to their related variables, thus, are not presented here.

### 3.4 Results from the LSTM model

**Fig 5:**
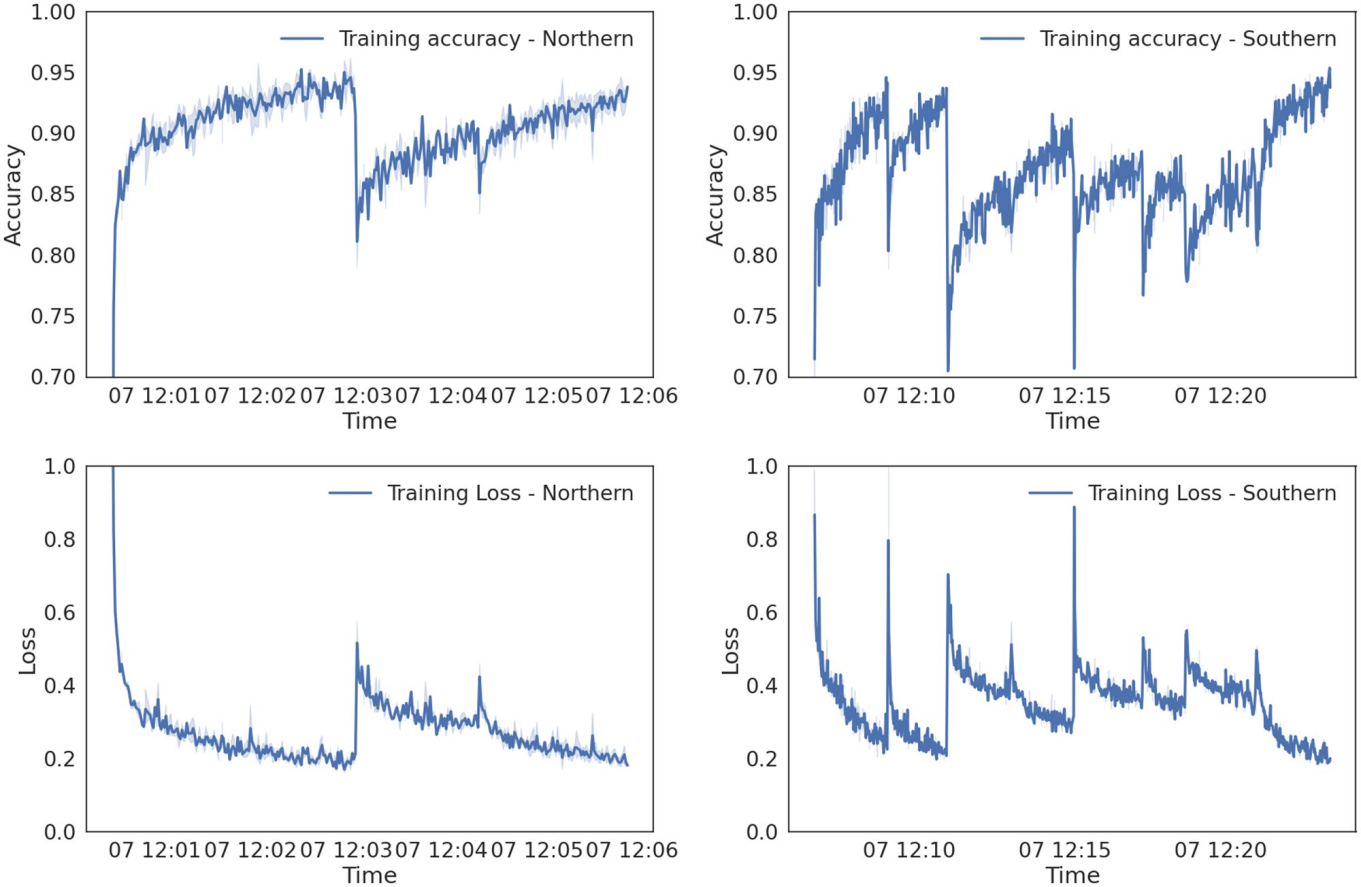
The training accuracy and loss metrics (top and bottom rows of panels, respectively) logged during the training procedures of the two LSTM neural networks, for the Northern (left column) and Southern clusters (right column) of federal regions. The jumps observed in both metrics indicate the point in time in which the callback function interrupted training on one federal region and carried on to the next.

As described in Section 2.7, two independent LSTM neural network models were trained, one for each of the two tick species investigated in this study. Data from the Northern cluster of federal regions were used to train the model for *I. persulcatus*, whereas data from the Southern cluster was used to train the model for *I. ricinus*. The training accuracy and loss metrics for the training procedures of the two models are shown in Fig 5. The time-series of the federal regions from the two clusters were used to train the models in a sequential series, i.e., the neural network was trained on the data of a federal region until training was interrupted by the callback function, the weights of the network were frozen, and training resumed on the data from the next federal region, until all the federal regions from the cluster were used. The jumps observed in the accuracy and loss metrics indicate the points in time where training on data from one federal regions was interrupted by the callback function and the procedure continued to the next.

**Fig 6:**
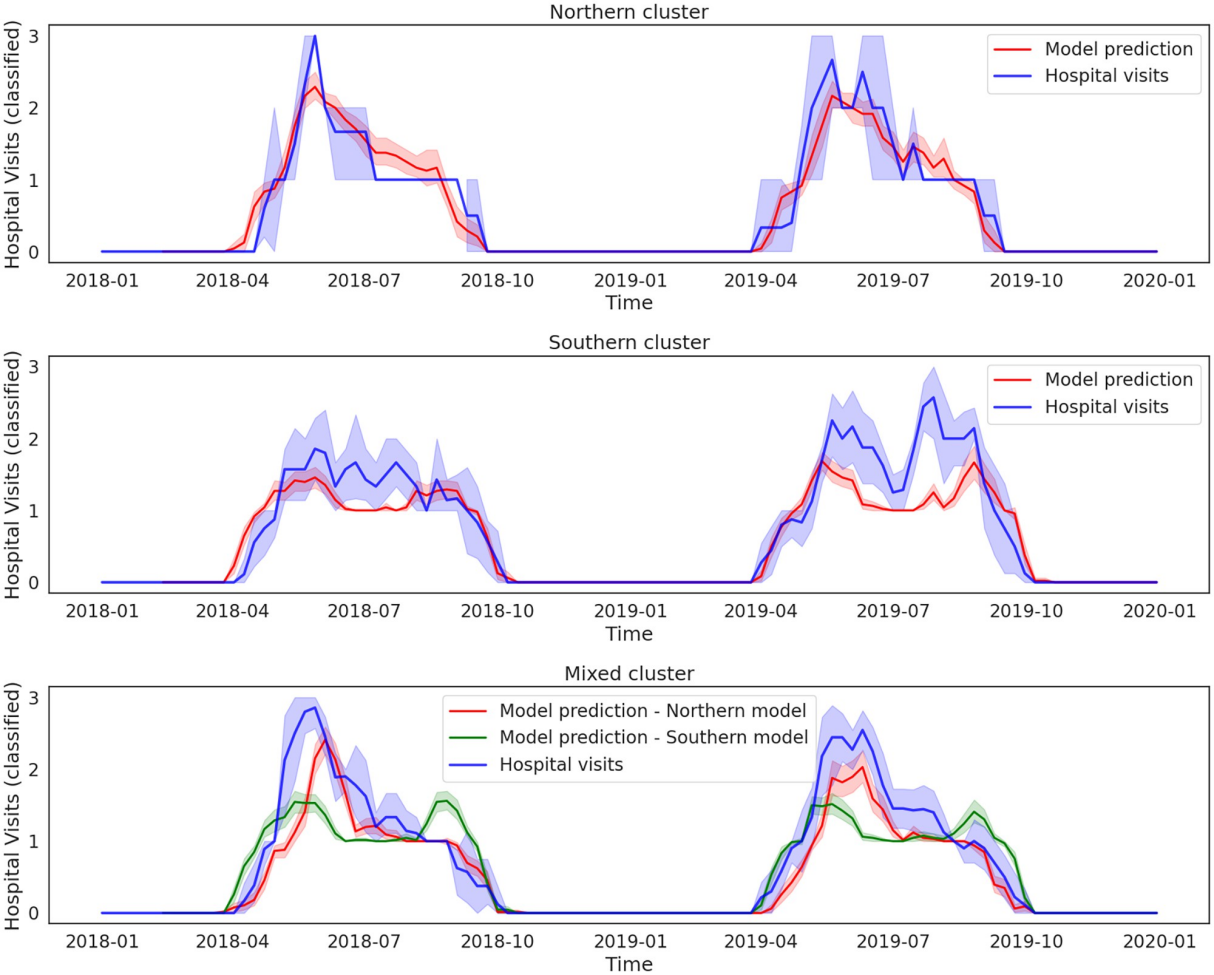
Model prediction and classified tick related hospital visits time series for the Northern cluster (top panel), Southern cluster (middle panel) and mixed cluster (bottom panel).

In Fig 6, the classified weekly hospital visits and the model prediction for the three clusters are shown. For the mixed cluster, both models were used to make predictions, as both species are found in these federal clusters. As described in the methods section, two independent models for each of the two tick species found in the Northern and Southern clusters of federal regions were trained and the mixed tick cluster was used as a test for the accuracy of the models. The two LSTM networks perform well in predicting the start and end of the tick season in the mixed cluster. The Northern model predicts the magnitude of the tick related hospital visits better for the mixed cluster, whereas the Southern model predicts a second smaller peak in the later stages of the summer period, which is not present in the hospital visits time series for this cluster of federal regions.

**Fig 7:**
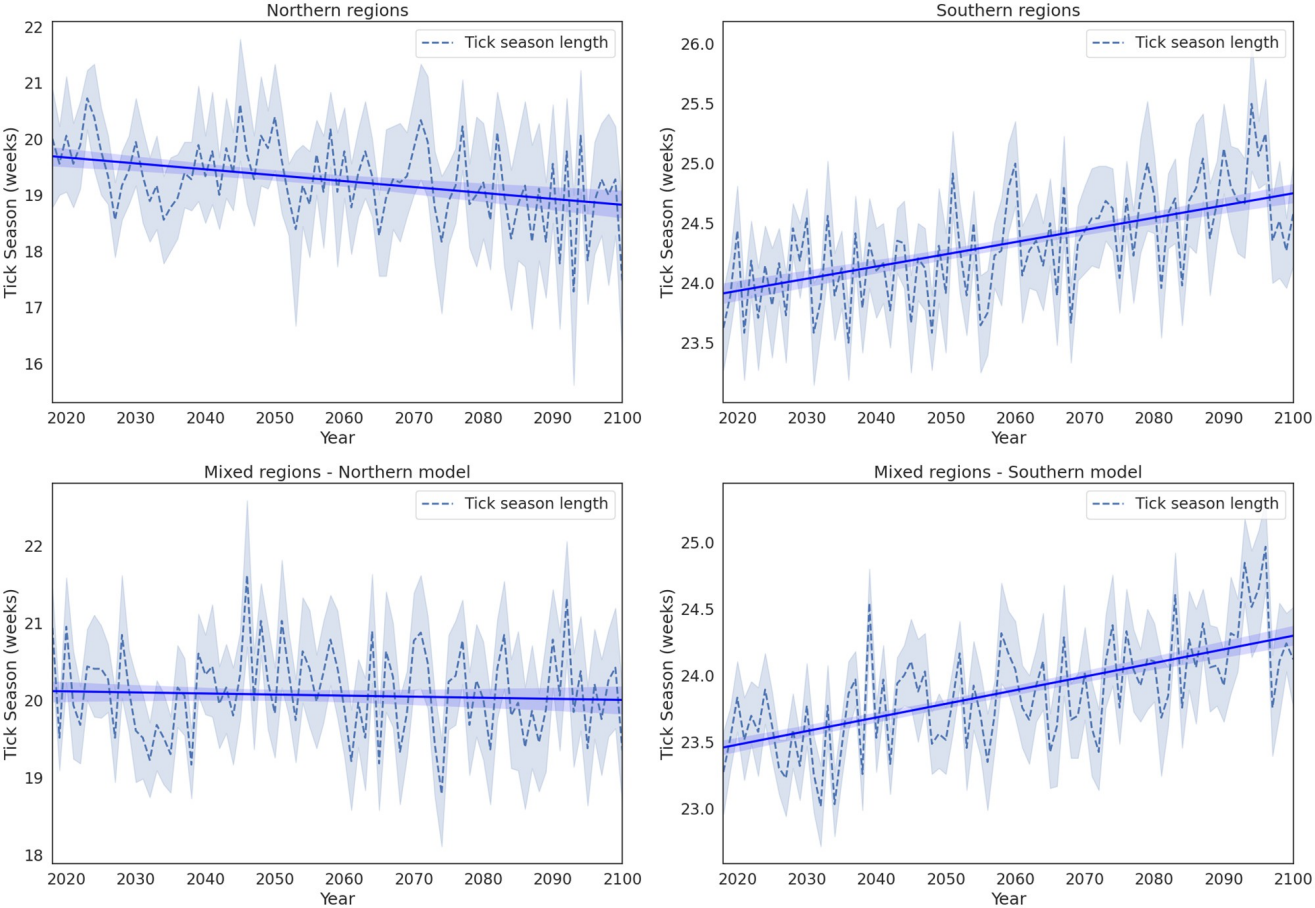
Tick season length predicted by the LSTM model for the Northern cluster (top left panel), Southern cluster (top right panel) and mixed cluster (bottom panels). For the mixed cluster, since both tick species are found there, both models were used to make predictions.

The trained LSTM models were then used to project the length of the tick season for the federal regions investigated in this study. In Fig 7, the average for each cluster is presented until the end of the 21st century, for the RCP8.5 climate scenario. The model projects a rather stable season length for the *I. persulcatus* tick, of around 20 weeks, until the end of the century. In the Northern cluster of federal regions, the tick season time series follows a slightly decreasing trend, whereas in the mixed cluster there is no discernible upward or downward trend. This observation is possibly due to the high dependence of the tick’s life cycle to photoperiod [2], which limits the effects of rising temperatures. For the *I. ricinus* tick, the model projections suggest an increase in tick season length, by about one week, until the end of the century in both the Southern and mixed clusters of federal regions.

**Fig 8:**
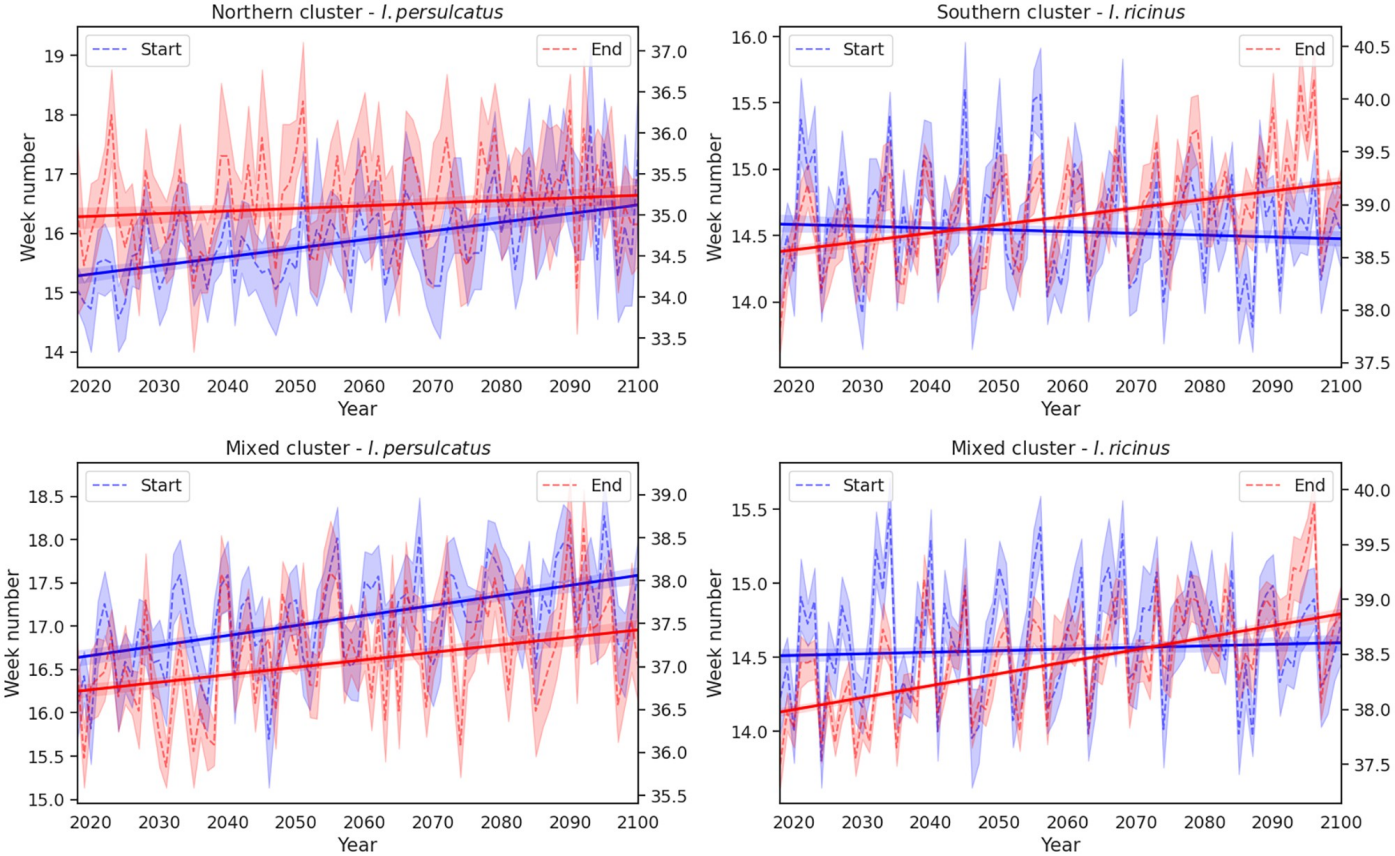
The start (blue) and end (red) of tick season projected by the LSTM models until the end of the century for the three federal region clusters investigated. The Southern cluster, where *I. ricinus* is located, is shown on the top right panel, the Northern cluster, where *I. persulcatus* is found, on the top left panel, and the mixed cluster, where both species of ticks are found, on the bottom row of panels. The dashed lines show the LSTM models’ projection in terms of week number and the solid lines a linear regression model fit to the time-series to visualise the trend.

To further investigate the tick season in the three clusters of federal regions, we examined the onset and end of tick season projected by the two LSTM models, shown in Fig 8 for the three clusters investigated in this study. In the Northern cluster, the slight decreasing tick season length is a result of the small, delayed onset of the season by about half a week, between the start and the end of the projection window. In the mixed cluster, there is a shift projected for the *I. persulcatus* tick season, as both the start and the end of the suitable window are observed to have an increasing trend. For the *I. ricinus* tick, the LSTM model projects lengthening of the tick season, which is attributed to the extension of the end of the season in both the Southern cluster and mixed cluster.

## 4 Conclusion

We investigated the link between climatic parameters and two different variables related to Ixodid ticks and tick-borne diseases in European Russia, namely tick-related hospital visits and borreliosis incidence rates. It is likely that both of the aforementioned incidence rates are underestimating the true numbers of tick-related hospital visits and borreliosis incidence rates, as it is probable that people will not seek medical care and, thus, these cases will go undocumented. The Rospotrebnadzor reports are however, to our knowledge, the most complete freely available records of such occurrences in European Russia’s federal subjects.

We found out that the federal regions in European Russia can be split into three clusters (Northern, Southern, and mixed) characterized by similar dominating tick species, similar dynamics of normalized borreliosis incidence rates and similar dynamics of normalized hospital visits. Interestingly, we detected a strong linear growth in the normalized incidence rates in the Southern cluster of the federal regions. In agreement with our results, growing trends in tick bites and borreliosis incidence rates have been reported for Tulskaya region [42], which is within Southern cluster. This is opposite to the expected growth in the north: although some increase in borreliosis incidence rates was observed between 1997 and 2003–2006, but then the curve levelled off. In line with these results, the tick-bite incidence rate also increased within approximately the same years in Komi and Arkhangelskaya federal regions, and then levelled off [9]. The data sets on encephalitis incidence rates in the aforementioned regions demonstrated increase starting in 1990–1994 to 2008–2010 [19, 20]. The difference with our results can be due to the different data set or due to the different disease considered as borreliosis is a bacterial disease, whereas encephalitis is viral. In the north and south, there are different encephalitis strains, with the northern strain being highly virulent [40]; thus, increase of encephalitis incidence rates can be expected.

For each of the clusters, we identified climatic variables with significant moderate correlations with borreliosis incidence rates. In the Northern cluster, this is the snowmelt date, in the Southern cluster, the temperature sums during warm season, and in the mixed cluster, the best correlation was found to be with two parameters: median temperature of the tick season and hydrothermal index. These climatic parameters explain from 25 to 40% of the variation in borreliosis incidence rates, depending on the cluster. This is a reasonable result given that there are many unknown factors that were not considered, including human behavior, land use change, host dynamics and others. Our results emphasize that the accuracy of prediction of incidence rates based on climatic parameters and using linear regressions is below 50%. Moreover, opposite to encephalitis, we did not find any association between increasing temperature and borreliosis incidence rates in the north of European Russia.

Furthermore, we investigated the correlations between the weekly dynamics of climatic parameters and weekly tick-related hospital visits. We show that all climatic parameters with pronounced seasonal cycle, such as air temperature, evaporation, vegetation indices and snow depth, show reasonably high correlations (maximum correlation coefficient between 0.7–0.8) with the hospital visits allowing for some time delays, which vary for different clusters of regions. This means a strong link between the ticks’ active season and these parameters and justifies usage of these parameters for modelling of hospital visits.

Finally, we trained two LSTM neural network models in the Northern and Southern clusters to predict hospital visits in the mixed cluster and to project future hospital visits until the end of the 21st century, under the RCP8.5 climate scenario. The model trained on the set of data from the Northern cluster showed better agreement with observed hospital visits in the mixed clusters. Overall, the model captured the magnitude of the tick-related hospital visits in the Northern and mixed clusters better, whereas two peaks in the Southern clusters were underestimated by the model. However, the tick season length was captured well by the two models for all clusters. Our projections show that the tick season length in the Northern and mixed clusters of federal regions does not change significantly until the end of the century, whereas in the Southern cluster, the season is prolonged by approximately one week. This change is expected to be due to earlier onset and later end of the season, in agreement with the current warming trends.

Based on our results, an increase in tick-related hospital visits and borreliosis incidence rates is expected in the southern forested areas of European Russia dominated by *I. ricinus*. This may sound as a counter-intuitive result because warming is amplified in the north and there is typically a higher fraction of *I. persulcatus* infected with borrelia. However, during the warm season, ticks undergo only one stage of development in the north, likely related to photoperiod [2]. Higher temperatures, thus, can only accelerate this one stage of development and even shorten, not prolongate the tick season. Moreover, we did not consider dispersal of ticks to the north and their establishment in the new areas which is the major expected change due to the warming climate. These trends proved to be hard to assess based on the climatic factors only. For example, in Arkhangelsk region, temperature and encephalitis incidence rates showed strong correlation but in Komi Republic, the same parameters showed a weaker correlation after 9-yr averaging [19, 20]. The reason might be that temperature is only an indirect factor, whereas it is the landscape change that facilitates vectors’ advancement [1]. These changes in the north solely due to warming can be slow, whereas faster changes can be induced by fires [43] or human activity. Particularly in the south, increasing temperature sums accelerate development of ticks on different stages, and ticks can undergo more than one stage of the life cycle during one warm season. This suggests a strong link between warming trends in southern regions and tick-related hospital visits and incidence rates, confirmed by our study.

## Supporting information

S1 Fig A schematic visualization of the training and prediction procedures implemented for projecting tick related hospital visits using the LSTM neural network. The input feature tensor consisted of the feature set of the last three weeks to account for the delayed effects of temperature and precipitation.
(TIF)

S2 Fig TLCC curves (top row of panels) and the time-series of the temperature at 2m and weekly tick-related hospital visits (bottom row) for the Southern (left panels), Northern (middle panels) and mixed (right panels) clusters of federal regions. (TIF)

S3 Fig TLCC curves (top row of panels) and the time-series of the evaporation and weekly tick-related hospital visits (bottom row) for the Southern (left panels), Northern (middle panels) and mixed (right panels) clusters of federal regions. (TIF)

S4 Fig TLCC curves (top row of panels) and the time-series of the snow depth and weekly tick-related hospital visits (bottom row) for the Southern (left panels), Northern (middle panels) and mixed (right panels) clusters of federal regions. (TIF)

S5 Fig TLCC curves (top row of panels) and the time-series of precipitation and weekly tick-related hospital visits (bottom row) for the Souther (left panels), Northern (middle panels) and mixed (right panels) clusters of federal clusters. The precipitation patterns in the three clusters did not vary significantly throughout the two years examined here, thus no significant cross-correlation coefficient was found between it and the weekly tick-related hospital visits time-series.
(TIF)

S1 Table List of meteorological stations used in this study. (PDF)

S2 Table The ERA5 variables used for performing time lagged cross correlation analysis between the tick related hospital visits and climate/land features. The minimum and maximum temperature features were derived from the 2m temperature hourly variable for each day.
(PDF)

S3 Table The variables used from the CMIP5 models for training the machine learning model and projecting tick related borreliosis incidence until the end of the 21st century. (PDF)

S4 Table The federal region clusters from where weekly tick bite related hospital visits were available. The clusters were divided into clusters with respect to the tick species and hospital visits dynamics found in them (Sec. 3.1).
(PDF)

S5 Table The federal region clusters from where yearly borreliosis cases rates were available. The clusters were divided into clusters with respect to the tick species found in them.
(PDF)
