# Correction: The impact of climatic factors on tick-related hospital visits and borreliosis incidence rates in European Russia

**DOI:** 10.1371/journal.pone.0322231

**Published:** 2025-04-04

**Authors:** Pantelis Georgiades, Ekaterina Ezhova, Meri Räty, Dmitry Orlov, Markku Kulmala, Jos Lelieveld, Svetlana Malkhazova, Kamil Erguler, Tuukka Petäjä

The following information is missing from the Funding statement: SM and DO are supported by RSF project No. 21-47-00016.
