# Supplementary figures and images for: The impact of climatic factors on tick-related hospital visits and borreliosis incidence rates in European Russia
Source: PLoS One. 2022 Jul 20;17(7):e0269846. doi: 10.1371/journal.pone.0269846 (PMC9299338; doi:10.1371/journal.pone.0269846)

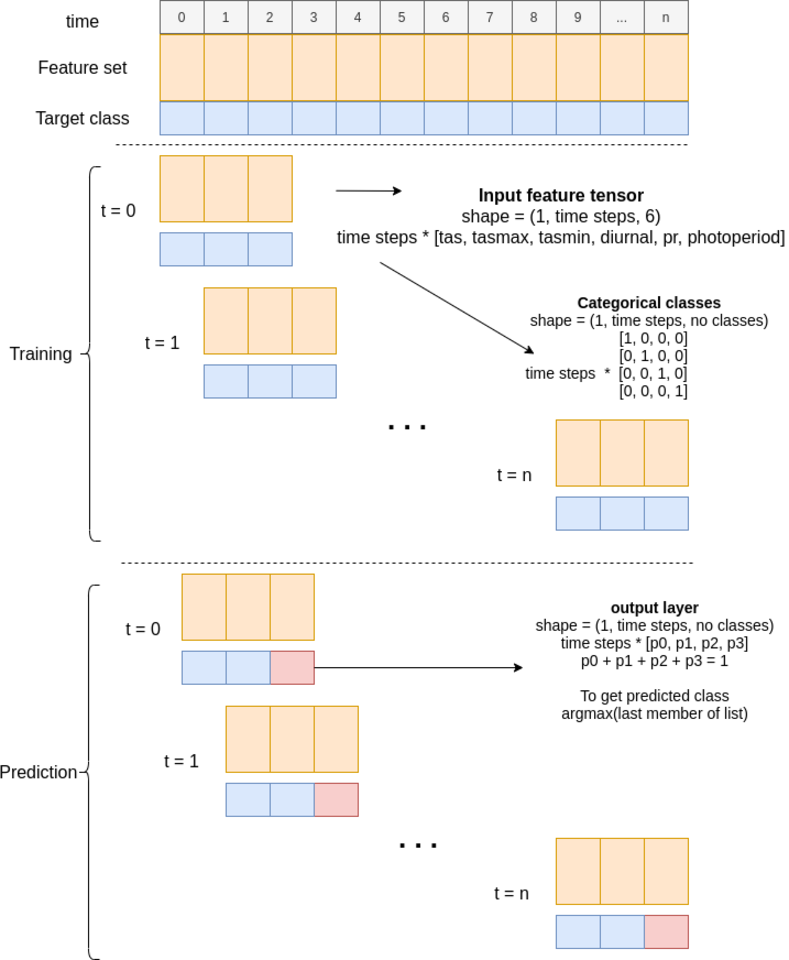

Supplement: S1 Fig — The input feature tensor consisted of the feature set of the last three weeks to account for the delayed effects of temperature and precipitation. (TIF) [file pone.0269846.s001.tif]

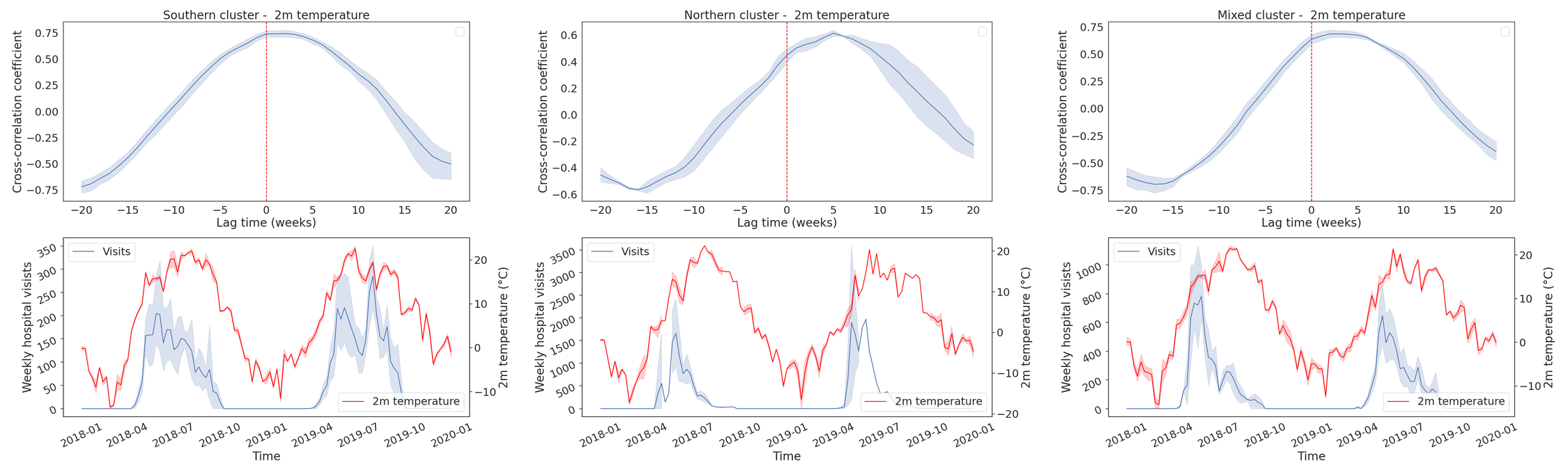

Supplement: S2 Fig — (TIF) [file pone.0269846.s002.tif]

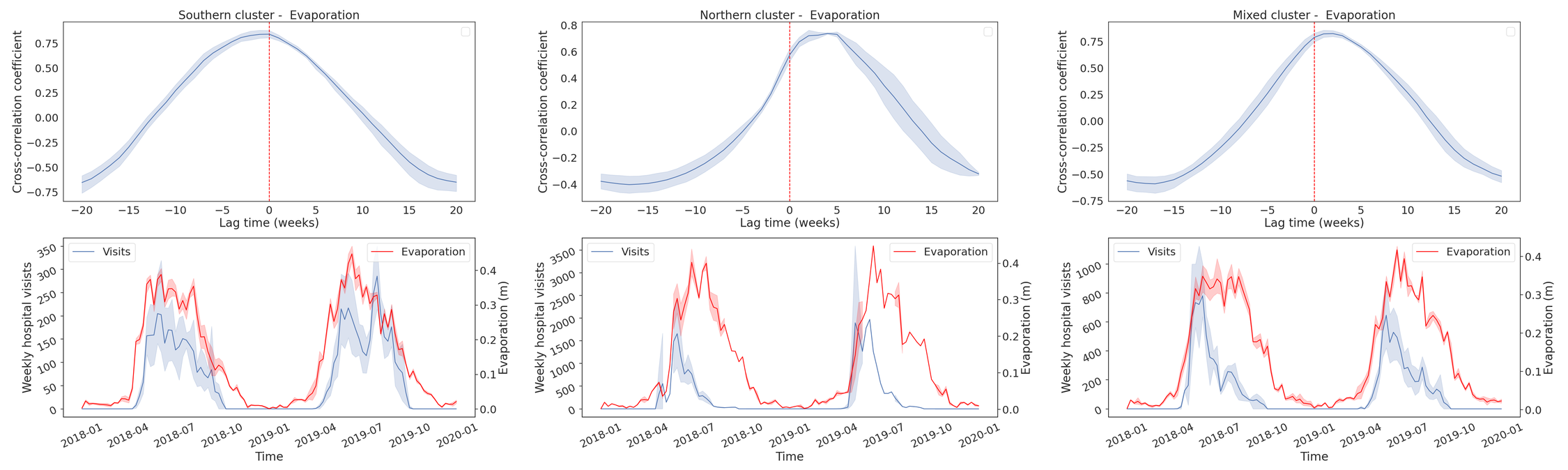

Supplement: S3 Fig — (TIF) [file pone.0269846.s003.tif]

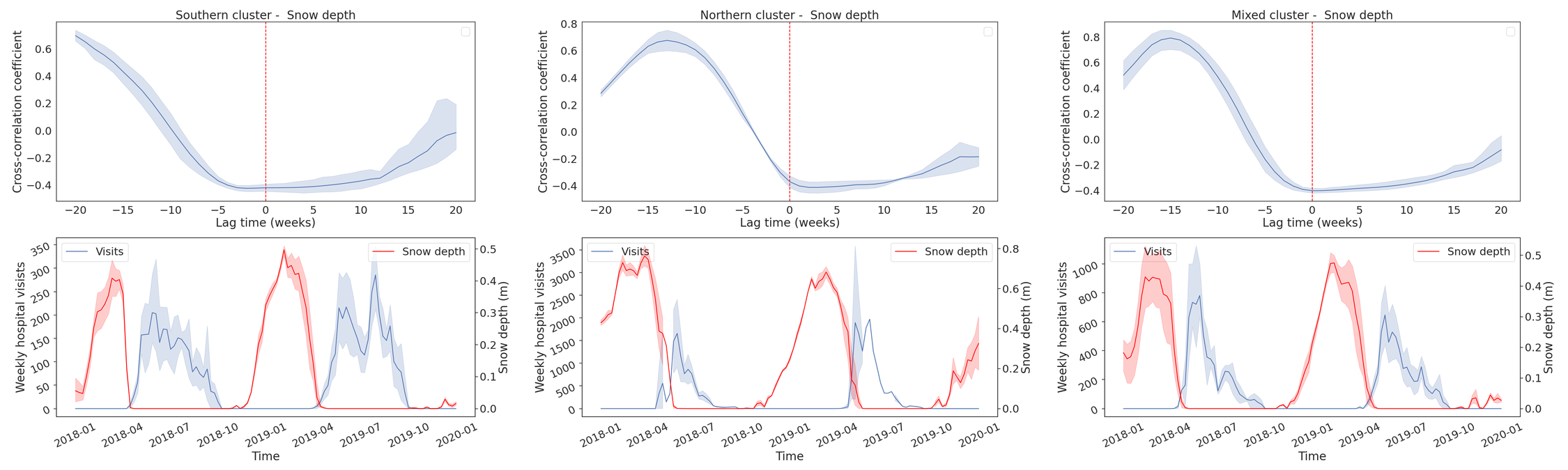

Supplement: S4 Fig — (TIF) [file pone.0269846.s004.tif]

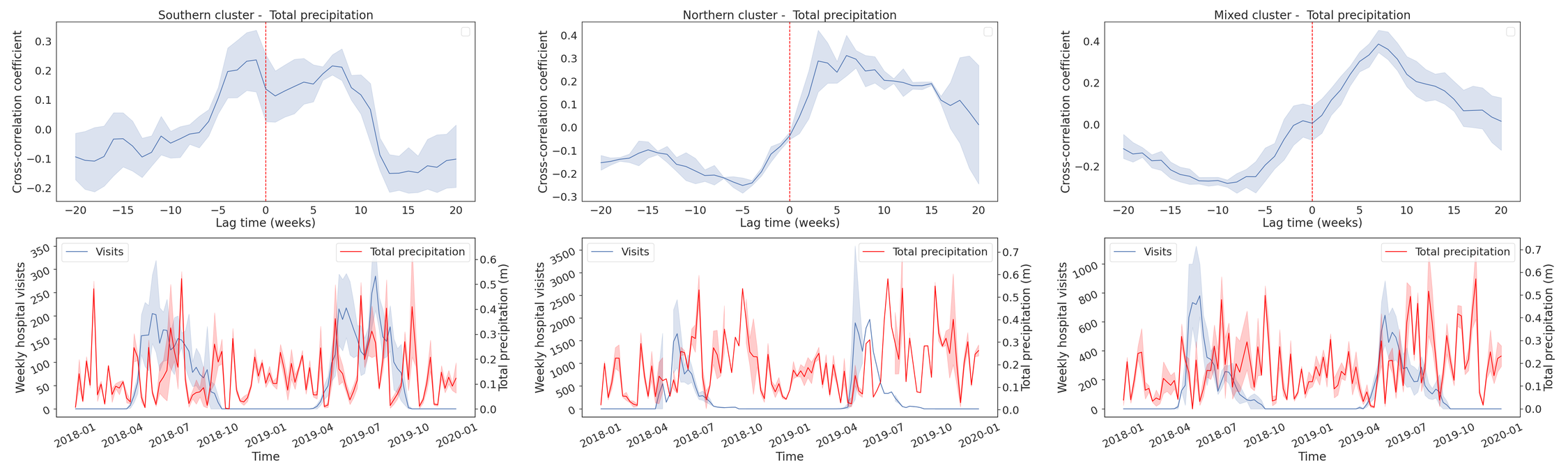

Supplement: S5 Fig — The precipitation patterns in the three clusters did not vary significantly throughout the two years examined here, thus no significant cross-correlation coefficient was found between it and the weekly tick-related hospital visits time-series. (TIF) [file pone.0269846.s005.tif]
