# Supplementary material for: The impact of climatic factors on tick-related hospital visits and borreliosis incidence rates in European Russia
Source: PLoS One. 2022 Jul 20;17(7):e0269846. doi: 10.1371/journal.pone.0269846 (PMC9299338; doi:10.1371/journal.pone.0269846)
Supplement: S1 Table — (PDF) [file pone.0269846.s006.pdf]

S1 Table **Table ST1** List of meteorological stations used in this study.

**WMO index**	**Station**	**Geographical position**		**Elevation, a.s.l.**
22408	Kalevala (Karelia)	65°13’N	31°09’E	118
22422	Gridino (Karelia)	65°54’N	34°46’E	9
22438	Zhizhgin (Karelia)	65°12’N	36°49’E	26
22520	Kem’ port (Karelia)	64°59’N	34°48’E	7
22602	Reboly (Karelia)	63°50’N	30°49’E	180
22802	Sortavala (Karelia)	61°43’N	30°43’E	17
22820	Petrozavodsk (Karelia)	61°49’N	34°16’E	110
22619	Padany (Karelia)	63°16’N	33°25’E	130
22471	Mezen’ (Arkhangelsk)	65°52¢N	44°13’E	14
22550	Arkhangelsk (Arkhangelsk)	64°30¢N	40°44’E	8
22583	Kojnas (Arkhangelsk)	64°45¢N	47°39’E	63
22641	Onega (Arkhangelsk)	63°54¢N	38°07’E	11
22676	Sura (Arkhangelsk)	63°35¢N	45°38’E	62
22768	Shenkursk (Arkhangelsk)	62°06¢N	42°54’E	40
22845	Kargopol’ (Arkhangelsk)	61°30¢N	38°56’E	124
22854	Nyandoma (Arkhangelsk)	61°40’N	40°11’E	232
22887	Kotlas (Arkhangelsk)	61°14’N	46°43’E	55
22837	Vytegra (Vologda)	61°01’N	36°27’E	55
22981	Velikij Ustyug (Vologda)	60°46’N	46°18’E	94
27008	Babaevo (Vologda)	59°24’N	35°56’E	136
27037	Vologda (Vologda)	59°19’N	39°56’E	125
27051	Tot’ma (Vologda)	59°53’N	42°45’E	134
27066	Nikolsk (Vologda)	59°32’N	45°28’E	142
22892	Vyborg (Leningrad)	60°43’N	28°44’E	10
22907	Gogland (Leningrad)	60°05’N	26°59’E	5
26063	St Petersburg (Leningrad)	59°58’N	30°18’E	3
26069	Belogorka (Leningrad)	59°21’N	30°08’E	88
26094	Tikhvin (Leningrad)	59°39’N	33°33’E	61
26157	Gdov (Pskov)	58°44’N	27°50’E	39
26258	Pskov (Pskov)	57°49’N	28°20’E	43
26359	Pushkinskie Gory (Pskov)	57°01’N	28°54’E	108
26477	Velikie Luki (Pskov)	56°21’N	30°37’E	104
27225	Rybinsk (Yaroslavl)	58°06’N	38°41’E	104
26275	Staraya Russa (Novgorod)	58°01’N	31°19’E	24
