# Supplementary material for: The impact of climatic factors on tick-related hospital visits and borreliosis incidence rates in European Russia
Source: PLoS One. 2022 Jul 20;17(7):e0269846. doi: 10.1371/journal.pone.0269846 (PMC9299338; doi:10.1371/journal.pone.0269846)
Supplement: S2 Table — The minimum and maximum temperature features were derived from the 2m temperature hourly variable for each day. (PDF) [file pone.0269846.s007.pdf]

S2 Table **Table ST2** The ERA5 variables used for performing time lagged cross correlation analysis between the tick related hospital visits and climate/land features. The minimum and maximum temperature features were derived from the 2m temperature hourly variable for each day.

**Short name**	**Long name**
d2m	2m dewpoint temperature
evaow	Evaporation from open water
fal	Forecast albedo
lai_lv	Leaf area index, low vegetation
lai_hv	Leaf area index, high vegetation
skt	Skin temperature
sde	Snow depth
sf	Snow fall
e	Evaporation
t2m	2m temperature
evavt	Evaporation from vegetation transpiration
pev	Potential evaporation
snowc	Snow cover
es	Snow evaporation
smlt	Snow melt
tp	Total precipitation
tasmin	Minimum temperature
tasmax	Maximum temperature
