# Supplementary material for: The impact of climatic factors on tick-related hospital visits and borreliosis incidence rates in European Russia
Source: PLoS One. 2022 Jul 20;17(7):e0269846. doi: 10.1371/journal.pone.0269846 (PMC9299338; doi:10.1371/journal.pone.0269846)
Supplement: S3 Table — (PDF) [file pone.0269846.s008.pdf]

S3 Table **Table ST3** The variables used from the CMIP5 models for training the machine learning model and projecting tick related borreliosis incidence until the end of the 21st century.

**Short name**	**Long name**
pr	Precipitation
tas	Mean temperature
tasmin	Minimum temperature
tasmax	Maximum temperature
diurnal	Diurnal temperature (max-min temperature)
