# Supplementary material for: The impact of climatic factors on tick-related hospital visits and borreliosis incidence rates in European Russia
Source: PLoS One. 2022 Jul 20;17(7):e0269846. doi: 10.1371/journal.pone.0269846 (PMC9299338; doi:10.1371/journal.pone.0269846)
Supplement: S4 Table — The clusters were divided into clusters with respect to the tick species and hospital visits dynamics found in them (Sec. 3.1). (PDF) [file pone.0269846.s009.pdf]

S4 Table **Table ST4** The federal region clusters from where weekly tick bite related hospital visits were available. The clusters were divided into clusters with respect to the tick species and hospital visits dynamics found in them (Sec. 3.1).

**Cluster**	**Federal regions**
North	Arkhangelskaya, Vologodskaya, Komi Rep.
South	Bryanskaya, Kaluzhskaya, Smolenskaya, Tulskaya, Ryazanskaya, Chuvashiya Rep., Mordoviya Rep., Ulyanovskaya
Mixed	Leningradskaya, Novgorodskaya, Pskovskaya, Tverskaya, Yaroslavskaya, Ivanovskaya, Kirovskaya, Kostromskaya, Mariy-el Rep., Permskaya, Udmurtiya Rep.
