# Supplementary material for: The impact of climatic factors on tick-related hospital visits and borreliosis incidence rates in European Russia
Source: PLoS One. 2022 Jul 20;17(7):e0269846. doi: 10.1371/journal.pone.0269846 (PMC9299338; doi:10.1371/journal.pone.0269846)
Supplement: S5 Table — The clusters were divided into clusters with respect to the tick species found in them. (PDF) [file pone.0269846.s010.pdf]

S5 Table Table ST5 The federal region clusters from where yearly borreliosis cases rates were available. The clusters were divided into clusters with respect to the tick species found in them.

**Cluster**	**Federal regions**
North	Arkhangelskaya, Karelya Rep., Vologodskaya
South	Bryanskaya, Kaluzhskaya, Ryazanskaya, Smolenskaya, Tulskaya
Mixed	Leningradskaya, Novgorodskaya, Pskovskaya, Tverskaya, Yaroslavskaya
